# Residual Adrenal Function in Autoimmune Addison’s Disease—Effect of Dual Therapy With Rituximab and Depot Tetracosactide

**DOI:** 10.1210/clinem/dgz287

**Published:** 2019-12-21

**Authors:** Catherine Napier, Earn H Gan, Anna L Mitchell, Lorna C Gilligan, D Aled Rees, Carla Moran, Krishna Chatterjee, Bijay Vaidya, R Andrew James, Yaasir Mamoojee, Simon Ashwell, Wiebke Arlt, Simon H S Pearce

**Affiliations:** 1 Institute of Genetic Medicine, International Centre for Life, Newcastle University, UK; 2 Institute of Metabolism and Systems Research, University of Birmingham, Birmingham, UK; 3 Neuroscience and Mental Health Research Institute, Cardiff University, Cardiff, UK; 4 University of Cambridge Metabolic Research Laboratories, Wellcome Trust-MRC, Institute of Metabolic Science, Addenbrooke’s Hospital, Cambridge, UK; 5 Royal Devon & Exeter Hospital, University of Exeter Medical School, Exeter, UK; 6 The James Cook University Hospital, Middlesbrough, UK; 7 NIHR Birmingham Biomedical Research Centre, University of Birmingham and University Hospitals Birmingham NHS Foundation Trust, Birmingham, UK

**Keywords:** Addison’s disease, regenerative medicine, immunotherapy, residual adrenal function, steroidogenesis, adrenocorticotropin

## Abstract

**Context:**

In autoimmune Addison’s disease (AAD), exogenous glucocorticoid (GC) therapy is an imperfect substitute for physiological GC secretion. Patients on long-term steroid replacement have increased morbidity, reduced life expectancy, and poorer quality of life.

**Objective:**

The objective of this article is to restore adrenocortical steroidogenic function in recent-onset AAD.

**Design:**

An open-label, multicenter trial of immunotherapy and trophic stimulation in new-onset AAD was conducted. Serial measurement of serum and urine corticosteroids at baseline and throughout a 72-week follow-up period was performed.

**Setting:**

This study was conducted at the

endocrine departments and clinical research facilities at 5 UK tertiary centers.

**Patients:**

Thirteen participants (9 female, 4 male; age 19-64 years) were included with AAD confirmed by high adrenocorticotropin, low circulating cortisol (basal < 100 nmol/L or post-tetracosactide < 300 nmol/L), and positive serum 21-hydroxylase antibodies.

**Intervention:**

All participants received dual therapy with B-lymphocyte–depleting immunotherapy (rituximab 1 g given twice) and repeated depot tetracosactide (1 mg on alternate days for 12 weeks).

**Main Outcome Measure:**

Restoration of normal GC secretion (stimulated cortisol > 550 nmol/L) at week 48 was the main outcome measure.

**Results:**

Ten of 13 (77%) participants had detectable stimulated serum cortisol (26-265 nmol/L) at trial entry. Following intervention, 7 of 13 (54%) had an increase in stimulated cortisol measurement, with a peak response of 325 nmol/L at week 18 in 1 participant. Increased steroid metabolites, assayed by urine gas chromatography–mass spectrometry at week 12 and week 48, was detected in 8 of 13 (62%) individuals, reflecting an increase in endogenous steroidogenesis. Four of 13 had residual adrenal function at 72 weeks.

**Conclusion:**

Combined treatment with rituximab and depot tetracosactide did not restore normal adrenal function. Nevertheless, adrenocortical plasticity is demonstrated in some patients, and this has the potential to be exploited to improve adrenal function.

Autoimmune Addison’s disease (AAD) is a rare disease in which immune-mediated destruction of steroid-producing cells in the adrenal cortex culminates in a potentially fatal state of steroid deficiency (1, 2). Steroid 21-hydroxylase and other adrenal steroidogenic enzymes are the target of immunological attack (3). Once levels of circulating glucocorticoids (GC) and mineralocorticoids (MC) fall to a critical state, patients are absolutely dependent on daily steroid replacement for survival.

The advent of cortisone acetate in the 1940s transformed the disease from certainly fatal to a manageable chronic condition. Nonetheless, synthetic GCs cannot mimic the intrinsic diurnal rhythm of cortisol production (4), thus current steroid replacement regimens are imperfect. Side effects from even a subtle excess of GC pose a risk to bone health, cardiovascular risk, and glucose tolerance (5-10). Despite regular steroid replacement, the risk of adrenal crisis remains an unpredictable and dangerous threat to health, and life expectancy is reduced in patients with AAD (11-13).

Adrenocortical plasticity has long been established (14), with several examples in clinical practice: patients receiving exogenous steroid therapy develop adrenal atrophy and functional adrenal failure; conversely, hypertrophy of the adrenal glands is seen in the setting of adrenocorticotropin excess (eg, Cushing’s disease). Recent early-phase studies of novel therapies have significantly advanced our understanding of the concept of residual adrenal function (RAF) in AAD and have suggested that adrenocortical plasticity may be amenable to intervention (15, 16). The use of B-cell–depleting immunotherapy in autoimmune disorders that share pathophysiological features with AAD has now translated to routine clinical care for some but not all conditions (17-19), and the first study in AAD treated 6 newly diagnosed patients with rituximab with some success (15). This B-lymphocyte–depleting anti-CD20 immunotherapy ameliorated the immunological destruction of steroid-producing cells in the adrenal gland in 1 patient—progressively increasing concentrations of endogenous GCs and MCs were seen, allowing a temporary complete cessation of replacement steroids (15). Thereafter, a second study of regenerative therapy in AAD was performed: thirteen patients with established AAD of longer than 1 year’s duration were treated with repeated doses of tetracosactide (adrenocorticotropin_1-24_, Synacthen Depot, manufactured by Mallinckrodt Pharmaceuticals). This trophic stimulation harnessed and exploited RAF in 2 patients (4 and 8 years from diagnosis)—levels of intrinsic GCs and MCs increased, and both patients stopped exogenous steroids entirely (16). One patient remains off steroid replacement 7 years later.

These early-phase studies have greatly enhanced our understanding of the potential of RAF and the impact regenerative medicine therapy could have in AAD. This paper reports on the RADS2 study, which combined therapy with B-lymphocyte–depleting immunotherapy and trophic adrenocorticotropin stimulation in newly diagnosed patients for the first time, with the aim of harnessing and exploiting endogenous adrenal steroidogenesis and ultimately delivering better outcomes for patients with this chronic disease.

## Patients and Methods

Thirteen individuals (9 female, 4 male; age 19-64 years) with a diagnosis of new-onset AAD within the preceding 4 weeks were recruited from endocrine or acute medical services in Newcastle, Exeter, Cambridge, or Cardiff, United Kingdom. Patients underwent robust clinical and biochemical screening at the point of trial entry to confirm unequivocally that adrenal failure was primary and of autoimmune origin. Eligibility criteria included age 10 to 65 years, clinical features to confirm primary adrenal failure, high adrenocorticotropin (> 47 ng/L), low circulating cortisol concentrations (basal < 100 nmol/L or stimulated 30 or 60 minutes post-tetracosactide < 300 nmol/L), and positive serum 21-hydroxylase antibodies (21OH Abs) (≥ 1 U/mL). A computed tomography scan and chest x-ray were also performed to exclude intercurrent illness or malignancy and to assess adrenal gland appearances. Exclusion criteria were significant cardiovascular or respiratory disease (including asthma), renal or hepatic disease, malignancy, pregnancy or breastfeeding, current infectious disease (including HIV, hepatitis B and hepatitis C, shingles/zoster, tuberculosis), unexplained abnormality on chest x-ray, and previous use of immunosuppressive or cytotoxic drugs (excluding GC).

**Figure 1.**
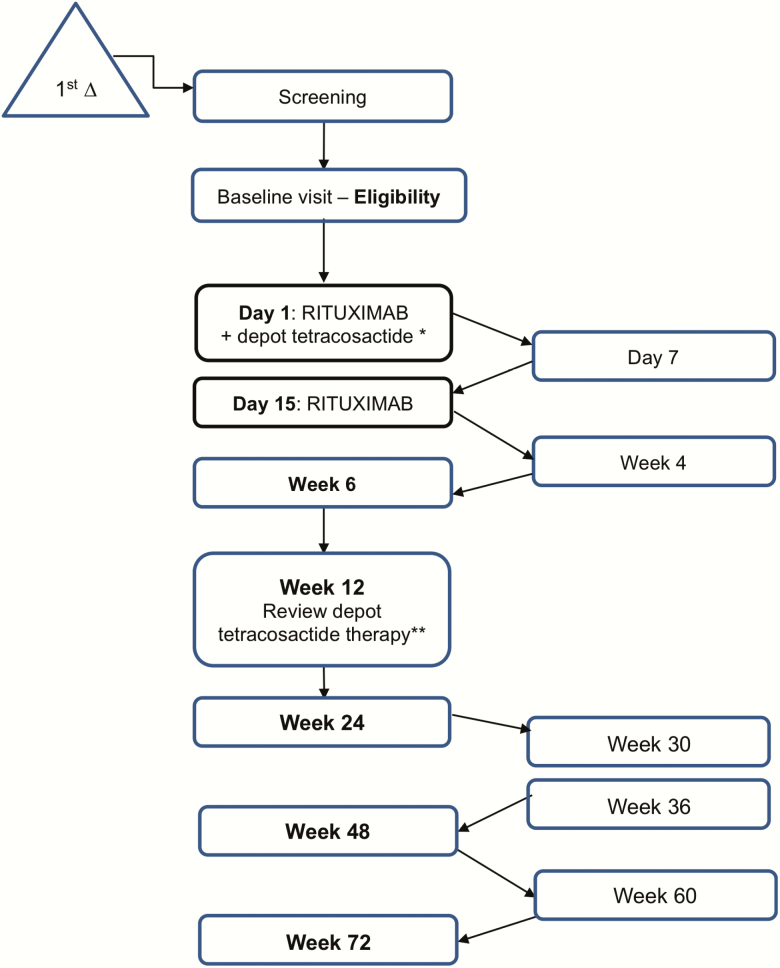
Screening, treatment and monitoring schedule in the RADS2 study. Initial screening was performed in person or by telephone or email. If a patient with primary adrenal failure who was willing to participate in the study was identified, then formal eligibility testing took place. Robust clinical and biochemical assessment was carried out at the study entry (baseline visit), before any intervention. Major outcome visits (shown in middle column) were performed with patients “free” of exogenous steroids, allowing assessment of endogenous steroid production. Interim safety visits (shown in right-hand column) allowed a shorter clinical assessment to take place, with the primary aim being patient safety. *Depot tetracosactide therapy started. One mg administered subcutaneously on alternate days. **Depot tetracosactide discontinued at week 12, or continued to a maximum of week 20, depending on response (increasing stimulated cortisol on week 6 and week 12 SST) and tolerability.

**Figure 2.**
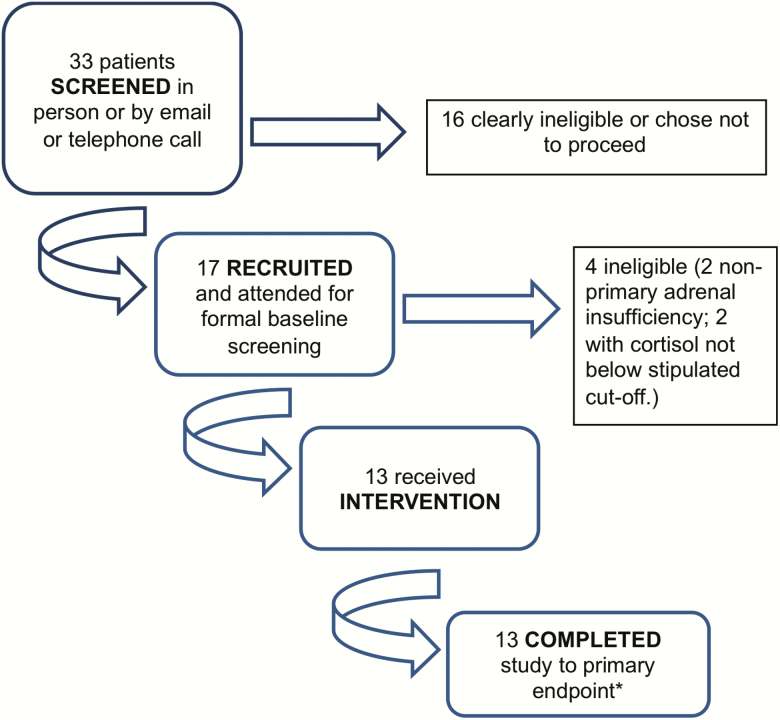
CONSORT flow diagram of patients screened, enrolled and treated in the RADS2 study. Thirty-three patients with adrenal failure diagnosed within the past 4 weeks were contacted by a member of the study team for discussion of the study and initial assessment of clinical history. Seventeen of 33 patients with primary adrenal failure who agreed to participate proceeded to formal consent and recruitment into the study. These patients were then formally screened to ensure they unquestionably met eligibility criteria. Four patients did not meet eligibility criteria on comprehensive assessment and could not proceed. Thirteen participants received treatment with rituximab and depot tetracosactide. Twelve participants completed 72 weeks of follow-up. *Primary endpoint assessed at 48 weeks = peak stimulated cortisol > 550 nmol/L

Thirty-three potential recruits were identified and underwent preliminary screening across 4 sites. Seventeen patients provided consent and were formally recruited into the 72-week study (Fig. 1). Of those who consented, 4 of 17 patients failed 1 or more eligibility criteria for treatment (Fig. 2).

The study was registered at ISRCTN with ID 20220821. Ethical approval was granted by the National Research Ethics Service North East-Sunderland, reference number 12/NE/0339.

### Design and intervention regimen

This open-label study of rituximab and depot tetracosactide followed newly diagnosed patients for 72 weeks after intervention to assess for any improvement in adrenocortical function. The schedule of visits for screening, intervention, and follow-up is outlined in Fig. 1.

Rituximab (1 g by intravenous infusion) was administered on day 1 and day 15 of the study. Patients were taught to self-inject 1 mg subcutaneous depot tetracosactide on day 1, and this was administered on alternate days for a minimum period of 12 weeks. In participants who had any biochemical evidence of an increasing stimulated-cortisol, tetracosactide was continued for a maximum period of 20 weeks in total.

At recruitment, all participants were taking hydrocortisone as GC replacement (in doses ranging between a total of 15 and 50 mg daily). A subset of patients had not yet commenced fludrocortisone, so this was started at the first clinical encounter with the trial team. GC replacement doses were lowered to a total daily dose of 10 to 15 mg hydrocortisone where possible to promote maximal endogenous adrenocorticotropin secretion. Throughout the study, in participants with a measurable improvement in endogenous steroidogenesis (any increase in basal or stimulated-cortisol concentrations), GC replacement was judiciously weaned, with regular monitoring of clinical symptoms, blood pressure, and serum electrolytes. The lowest daily GC replacement dose reached was 5 mg hydrocortisone daily in 1 patient.

### Outcome measures and assessments

The primary outcome measure was restoration of normal GC secretion at week 48, defined as a peak stimulated cortisol of greater than 550 nmol/L. Secondary outcome measures were restoration of normal GC secretion at weeks 6, 12, 24, and 72, improvement of basal or peak cortisol (>100 nmol/L over baseline), changes of other biochemical parameters (dehydroepiandrosterone sulfate [DHEA-S] and 17-hydroxyprogesterone [17OHP]), and the safety and tolerability of the regimen.

Participants had regular follow-up during the first 24 weeks of the study, with clinical assessment ± biochemical assessment on day 1, 7, 14, and 28 and then at 6, 12, 18, and 24 weeks. Major outcome visits included a short Synacthen test (SST) and detailed serum biochemistry sampling and were performed at week 6, 12, 24, 48, and 72, during a 36-hour “steroid medication–free” window to allow assessment of endogenous steroid production (Fig. 1). Overnight urine collections were performed during the steroid-free window and a comprehensive panel of urine steroids was measured at baseline, week 12, and week 48. Participants underwent robust education and overnight hospital admission while steroid free to ensure safety. In the event of intercurrent illness, the visit was postponed for a short time; in one instance, a visit was canceled. Electrolytes, full blood count, lymphocyte subsets, and SSTs (off replacement steroids) were analyzed in real time. All other blood samples and urine collections were stored at –80°C and batch-analyzed on trial completion.

Flow cytometry analysis of B-lymphocyte subsets was performed on fresh material throughout the study to assess the depth of B-lymphocyte depletion. CD19+ cells were measured at baseline and following intervention; 10 000 lymphocyte events were counted twice at each measurement. Complete depletion was judged as CD19+ less than 0.1% of lymphocytes.

SSTs (with cortisol measured by competitive chemoluminescent assay, lower limit of detection[LLD] 24 nmol/L) were performed at baseline, 6, 12, 24, 48, and 72, weeks and processed centrally with analysis performed in real time. A total of 250 µg soluble Synacthen (adrenocorticotropin_1-24_) was administered intramuscularly following a baseline blood sample drawn for cortisol measurement, with further samples drawn at 30 and 60 minutes. Before SST, a series of serum and plasma samples were drawn and stored to allow batch analysis of adrenocorticotropin (solid-phase, chemoluminescent assay, LLD = 5 ng/L), dehydroepiandrosterone sulfate (DHEA-S; solid-phase competitive chemoluminescent assay, LLD = 0.1 μmol/L), androstenedione (solid-phase competitive chemoluminescent assay, LLD = 1.05 nmol/L), aldosterone (solid-phase radioimmunoassay, LLD = 70 pmol/L), 17-hydroxyprogesterone levels (17OHP; radioimmunoassay, LLD = 1 nmol/L), and 21OH Abs (enzyme-linked immunosorbent assay kit from RSR Ltd; positive result ≥ 1.0 U/mL) (20).

A comprehensive panel of urine steroids, collected in the steroid medication–free window, were measured at baseline, week 12, and week 48: GC precursors, GC metabolites, MC precursors, MC metabolites, and androgens were measured by gas chromatography–mass spectrometry in the laboratory at the Steroid Metabolome Analysis Core, Institute of Metabolism and Systems Research, Birmingham, UK. Thirty-two individual urinary steroids was quantified on an Agilent 5975 instrument after free and conjugated steroids were extracted from 1 mL of urine by solid-phase extraction (21). Urine metabolomic results were corrected for collection duration. No female patients were taking the oral contraceptive pill or hormone replacement therapy during the urine sample collections.

One major outcome visit was missed entirely because of illness (unsafe to stop steroid-replacement medication), and other safety visits were delayed by several days because of unavoidable commitments that participants could not reschedule. This was an uncontrolled exploratory study and descriptive statistics are used to present outcome measurements. Where appropriate, continuous variables were analyzed by paired t tests.

## Results

### Participant baseline characteristics

Twelve of 13 participants (mean age 44 years; range, 19-64 years) reported they had experienced weight loss, nausea or vomiting, and postural symptoms prior to diagnosis. Eleven of 13 reported salt craving, and all 13 described fatigue or lethargy. Eleven of 13 were pigmented and 9 of 13 were in “crisis” at the point of diagnosis (with adrenal crisis defined as requiring hospital admission for parental steroids and intravenous fluids). Seven of 13 had concurrent autoimmune diseases (hypothyroidism [n = 5], pernicious anemia [n = 2], Graves disease [n = 1], and premature ovarian failure [n = 1]), with 1 participant having a triad of autoimmune hypothyroidism, pernicious anemia, and premature ovarian failure (Table 1).

**Table 1.** Clinical and biochemical characteristics at baseline

Participant	Age, y	Sex	Baseline Stimulated Cortisol, nmol/L^a^	Adrenocorticotropin at Study Entry, ng/L	21OH Antibodies at Study Entry, U/mL	Other Autoimmune Disease
1	45	F	40	1085	6.0	
2	64	F	< 24	68	22.3	GD
3	36	F	< 24	1050	39.6	PA
**4**	**56**	**F**	265	316	5.5	AH
5	24	F	55	827	3648	
**6**	**56**	**F**	26	915	11.6	AH
7	43	F	< 24	850	71.7	AH
8	27	M	30	1054	413.3	
9	19	M	40	1542	581.7	
**10**	**48**	**M**	145	535	10.8	
11	60	F	45	1160	63.4	AH, PA, POF
12	39	F	88	393	17.0	AH
**13**	**52**	**M**	81	2630	2.8	

Thirteen treated participants. Mean age 44 years (range, 19-64 years). Stimulated cortisol on short Synacthen test at study entry ranges from less than 24 to 265 nmol/L; peak value of 30 or 60 minutes post-tetracosactide is shown. Demographic features of those who had serum cortisol 99 nmol/L or greater at 72 weeks are highlighted in bold.

Abbreviations: 21OH, 21-hydroxylase; AH, autoimmune hypothyroidism; F, female; GD, Graves’ disease; M, male; NR, normal range; PA, pernicious anemia; POF, premature ovarian failure.

^a^To convert serum cortisol values to μg/dL, divide by 27.6. Adrenocorticotropin at baseline ranged between 68 and 2630 ng/L (NR 0-47 ng/L), and all 12 participants had positive 21OH antibodies (2.8-3648 U/mL; NR < 1 U/mL).

Ten of 13 participants had detectable but subnormal stimulated cortisol on SST at formal screening at trial entry (26-265 nmol/L). All had elevated adrenocorticotropin levels (68-2630 ng/L; normal range [NR] 0-47 ng/L) and positive 21OH Abs (2.8-3648 U/mL; NR < 1 U/mL) (Table 1).

### Adrenal steroidogenic function: serum

**Figure 3.**
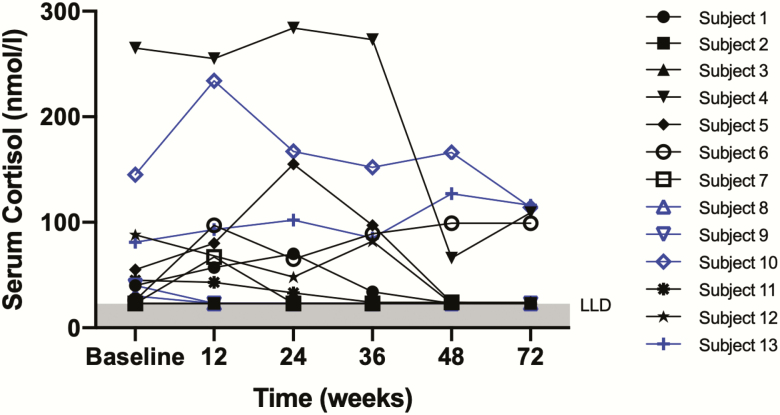
Peak stimulated cortisol on short Synacthen testing at baseline and major outcome visits. Peak stimulated cortisol (higher value of 30 or 60 minutes post-tetracosactide) at baseline (study entry) and at each major outcome visit: week 6, 12, 24, 48 (primary outcome assessment), and 72. Seven of 13 participants had an increase in stimulated cortisol recorded on 1 or more follow-up visits. Four participants (4, 6, 10, and 13) completed the study with a stimulated cortisol measurement of 99 nmol/L or greater. Male participants are shown in blue. Lower limit detection = 24 nmol/L.

Seven of 13 participants demonstrated an increase in endogenous cortisol following intervention (an increase in stimulated cortisol on SST detectable during sampling from at least 1 major outcome visit; *P* = .45 at week 48 vs baseline visit; paired t test) (Fig. 3). No participants met the primary study outcome with restoration of normal endogenous steroidogenesis (stimulated cortisol > 550 nmol/L), but 1 participant (participant 5) did achieve a secondary outcome measure with an increase in stimulated cortisol from 55 nmol/L to 155 nmol/L following intervention. This female patient (age 24 years) had clear evidence of sustained endogenous steroidogenesis following rituximab therapy and adrenocortical stimulation. Of note, this patient had the highest titer of 21OH Abs at trial entry (3648 U/mL) and the longest duration of symptoms prior to diagnosis (fatigue and hyperpigmentation of several years’ duration).

Participant 4 had the highest recorded serum cortisol during the study—284 nmol/L post-Synacthen at week 12 (an early morning cortisol measurement taken as part of safety surveillance at week 18 was 325 nmol/L). This 56-year-old woman retained clear evidence of endogenous steroidogenesis for more than 12 months after trial entry.

Participant 10 did not meet any biochemical end points but did have noteworthy endogenous function throughout the study. At trial entry, his peak cortisol was 145 nmol/L, with a significant increase to a peak cortisol of 234 nmol/L at week 6. Depot tetracosactide was continued for 20 weeks—he retained detectable endogenous steroidogenesis at week 72 (peak cortisol 114 nmol/L on stimulation). Another male patient (participant 13) maintained endogenous steroidogenesis throughout the 72-week follow-up period with a stimulated cortisol at trial entry of 81 nmol/L, 127 nmol/L at week 48, and 116 nmol/L at week 72. At week 72, 4 of the 13 (31%) participants had stimulated serum cortisol concentrations of 99 nmol/L or greater, suggesting residual adrenal function. These 4 individuals had higher mean serum cortisol at baseline than the rest of the cohort (129 nmol/l vs 41 nmol/l; *P* = .03) but were not different with regard to other baseline characteristics.

**Figure 4.**
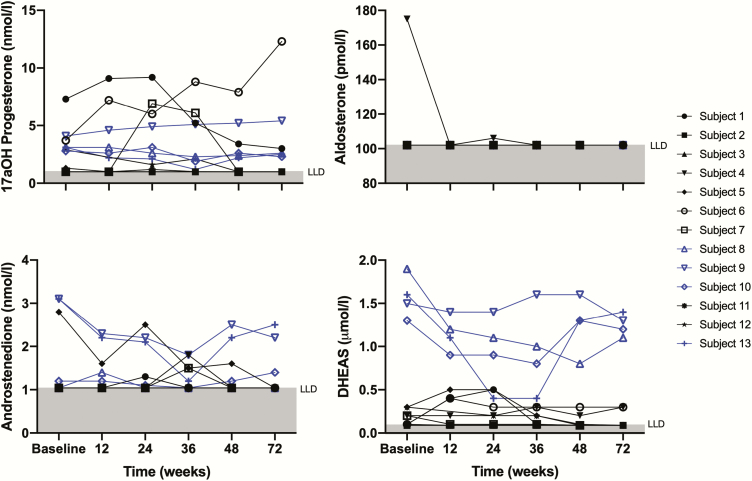
Serum steroid biochemistry at baseline and major outcome visits. DHEA-S (µmol/L; solid-phase competitive chemoluminescent assay, lower limit detection [LLD] = 0.1 µmol/L), 17aOHP (nmol/L; radioimmunoassay, LLD = 1 nmol/L), aldosterone (pmol/L; solid-phase radioimmunoassay, LLD = 70 pmol/), and androstenedione (nmol/L; solid-phase competitive chemoluminescent assay, LLD = 1.05 nmol/L) were measured in all treated participants at each major outcome assessment (baseline, week 6, week 12, week 24, week 48, and week 72). Samples were collected before each major outcome visit SST, and batch analysis was performed on trial completion. Gray shading denotes LLD for each steroid measured. Male participants are shown in blue. Participants with the highest levels of DHEA-S are all male, likely representing a testicular source of DHEA-S. No patients were taking DHEA supplementation.

Measurements of DHEA-S, androstenedione, aldosterone, and 17OHP in serum are shown in Fig. 4. Aldosterone was undetectable throughout in all patients, except at baseline and at week 12 in participant 4, who had the highest recorded stimulated cortisol in the study. 17OHP was higher in female participants, reflecting the contribution of an ovarian source. Similarly, serum DHEA-S concentrations were higher in men.

### Adrenal steroidogenic function: urine

**Figure 5.**
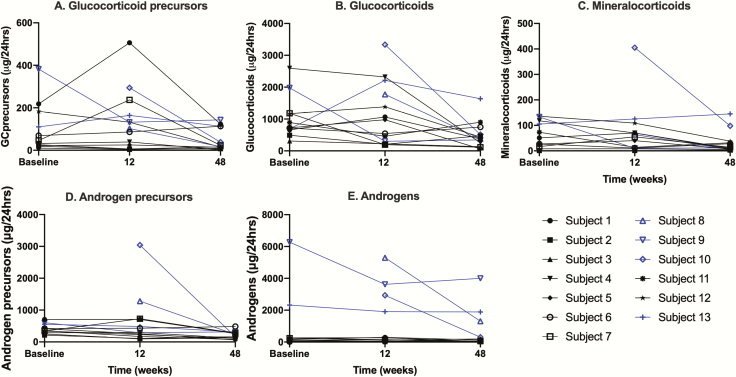
Urine steroid metabolite excretion (µg/24 hours) at baseline, week 12, and week 48. A comprehensive urine steroid profile was measured by gas chromatography–mass spectrometry at baseline, week 12 and week 48 (primary outcome assessment) of the study (see “Methods”). A, The sum of the metabolites of the glucocorticoid (GC) precursors (17-hydroxyprogesterone, 17-hydroxy-pregnanolone, pregnanetriol) and 11-deoxycortisol (tetrahydro-11-deoxycortisol) are plotted. B, The sum of the active GC metabolites (cortisol, tetrahydrocortisol, 5α- tetrahydrocortisol, α-cortol, β-cortol, cortisone, tetrahydrocortisone, α-cortolone, and β-cortolone) are shown. C, The sum of the mineralocorticoid metabolites (3α,5β-tetrahydroaldosterone, tetrahydrocorticosterone, 5α-tetrahydrocorticosterone, tetrahydrodeoxycorticosterone, 5α-tetrahydrodeoxycorticosterone, tetrahydro-11-dehydrocorticosterone, and 5α-tetrahydro-11-dehydrocorticosterone) are plotted. D, The sum of the androgen precursor metabolites dehydroepiandrosterone, 16α-dehydroepiandrosterone, 5-pregnanediol, and 5-pregnanetriol are plotted. E, The sum of the major active androgen metabolites androsterone and etiocholanolone are plotted over time. Total urine GC metabolite production (shown here; estimated production in µg/24 hours) increases between study entry and week 12 in 4 of 13 participants (1, 5, 12, and 13). Three of 13 participants (4, 10, and 13) with stimulated cortisol 100 nmol/L or greater at week 72 had the highest levels of GC metabolite production in urine. Baseline urine samples from participant 8 and participant 10 were not available.

Eight of 13 participants demonstrated increasing urinary steroid metabolite excretion postintervention, indicating an increase in endogenous adrenal steroidogenesis. In these 8 individuals, we saw a pattern of increased GC precursor excretion, notably 17-hydroxypregnanolone, pregnanetriol, and tetrahydro-11-deoxycortisol, during the first 12 weeks of the study in several participants, with a subsequent decline by week 48. Total GC metabolite production followed a similar pattern in a smaller numbers of participants (4/13), with an increase in urinary GC excretion between baseline and week 12 (Fig. 5). Three participants (4, 10, and 13) who maintained a peak serum cortisol greater than 100 nmol/L at week 72 had excreted the highest amounts of GC metabolites among all participants (Fig. 5). A fourth individual (participant 1) had significant changes in urinary steroid output, with increases in a range of GC precursors (pregnanediol, 17hydroxy-pregnanolone, pregnanetriol, pregnanetriolone, and tetrahydro-11-deoxycortisol). This increase in production of steroid precursors indicates an authentic steroidogenic response to adrenocorticotropin stimulation. Four of 13 participants had an increase in total MC metabolite production from baseline to week 12 and 5 of 13 participants had an increase in androgen metabolite production during the same period when comparing preintervention and postintervention. Pooling results from all the participants, there was no statistically significant increase in excretion of urine steroid metabolites between baseline and week 48.

In terms of correlation between serum and urine steroid response, 7 of 13 participants had a detectable serum response (any increase in stimulated cortisol from baseline), whereas 8 of 13 individuals had a detectable increase in urine steroid excretion (overlapping with 6 of the serum responders). Notably, the number and range of increasing urinary steroid metabolites excreted posttreatment is not necessarily reflected in the serum steroid response. For example, participant 1 had only a small increase in peak stimulated cortisol following intervention (increment of 30 nmol/L in stimulated cortisol measurement at week 12), but demonstrated increased urinary steroid metabolite excretion across the spectrum including GCs, GC precursors, MCs, androgens, and androgen precursors.

### Immune parameters

At baseline, 21OH Ab titers ranged from 2.8 to 3648 U/mL (positive result ≥ 1.0 U/mL). Serum immunoglobulin M levels fell over the course of the study, but remained within reference range (22). Twelve of 13 participants achieved CD19+ counts measured as 0.0 or 0.1% of the lymphocyte population following immunotherapy, with counts remaining low for several months (minimum of 12 to a maximum of 48 depleted weeks). Resurgence (> 0.5% of lymphocyte population) was detectable in all participants by the end of the study and occurred after a median period of 48 weeks (range, 12-72 weeks) (22). Participant 8 did not achieve complete CD19+ depletion (lowest count 0.2% at week 6) and did not have an increase in stimulated cortisol following intervention.

### Safety and tolerability

Trial medications were well tolerated by all participants. All infusions of rituximab were completed. All patients completed the active treatment phase; 1 of 13 did not complete the 72-week follow-up period (attended until week 48, did not attend final week 72 visit). Localized reactions to tetracosactide were frequently reported—redness, swelling, and bruising around abdominal injection sites (these reactions have been previously reported with repeated doses of tetracosactide) (16, 23).

Four serious adverse events were recorded during the study; none were found to be causally related to the study interventions (22). Multiple adverse events were recorded across all sites during the study; frequently reported symptoms or minor illnesses were headaches, back pain, sore throat, and sinusitis.

## Discussion

Recent early-phase experimental studies of novel therapies have enabled small numbers of patients with AAD to wean and stop steroid replacement following intervention, transforming a chronic disease into a potentially curable condition (15, 16, 24): This heralds an opportunity for a transformation in AAD management. The aim of this study was to combine immunotherapy and trophic stimulation to harness RAF, aiming to regenerate steroidogenic function, with the hope that dependence on steroid replacement and patient outcomes could improve further with dual therapy. It was anticipated that increases in serum and urine GC postintervention would reflect improving endogenous steroidogenesis, with these biochemical changes potentially mirrored by enhanced quality of life indicators and fewer adrenal crises—ultimately less morbidity and a reduced disease burden for patients.

No participants met the primary study outcome of restoration of endogenous steroidogenesis demonstrated by a stimulated cortisol greater than 550 nmol/L; nevertheless, 54% (7/13) did demonstrate an increase in serum cortisol postintervention. The highest serum cortisol midstudy was achieved by participant 4 (stimulated cortisol was 284 nmol/L at week 12): This allowed GC replacement to be weaned to 5 mg hydrocortisone per day for 5 months. Participant 5 achieved a secondary outcome measure with an increase in serum cortisol of 100 nmol/L, and 4 others (4, 6, 10, and 13) had sound biochemical evidence of maintained RAF, manifest as serum cortisol of 99 nmol/l or more at 72-week follow-up. Although pathophysiologically interesting, this residual steroidogenic function is not of the magnitude required to make a difference to the clinical well-being or hormone replacement of these patients. Furthermore, because there was no control group, we cannot exclude that the low-level persisting adrenal function was caused by the spontaneous natural history of the condition and unrelated to the trial medication.

Urine gas chromatography–mass spectrometry did herald meaningful results, with 62% (8/13) demonstrating an increased excretion of urinary steroid metabolites postintervention. This pattern of urinary steroid excretion was variable between participants, but 1 (participant 1) had a demonstrable increase across panels of GC metabolites, GC precursors, and androgens. Importantly, the increase in steroid precursor metabolites unequivocally indicates improving endogenous adrenocortical function. Although the numbers of participants in this study, and therefore those with a detectable positive response, are low, this is objective evidence of improving RAF many months after a proven diagnosis of AAD. In the previous study using adrenocorticotropin_1-24_ stimulation (16), analysis of urine steroid profiles in the 2 participants who responded revealed that urinary excretion of GC precursors and active GC metabolites gradually increased from less than the fifth centile to greater than the median of healthy female controls at 10 weeks (in 1 responder) and at 40 weeks (in a second responder), with a parallel increase in urine MC metabolite excretion. In both patients, androgen precursor and active androgen metabolite excretion was slower to increase.

In the present study, when urine response is compared to levels of steroids detected in serum, it is apparent that serum steroid measurements cannot provide a comprehensive illustration of endogenous gland function. Therefore, assessment of urinary steroid excretion should be considered the most reliable method of analysis of endogenous steroidogenesis in future studies: Urine steroid assays are our most valuable and robust tool for appraising adrenal gland function.

Around 40% of patients with AAD have never been hospitalized with an adrenal crisis (25, 26); perhaps a reduced risk of crisis might be correlated to detectable RAF, which will have a protective effect. The 2 participants who responded to adrenocorticotropin stimulation in the previous early-phase study had never been hospitalized with an adrenal crisis (16). In the present study, participant 4 presented with a crisis (defined by hospital admission with requirement for parenteral steroids and intravenous fluids), but participant 5 did not. Clearly there are insufficient data in this study to predict how protective RAF may be, but it does warrant further exploration because emerging data suggest it is not rare among patients with established AAD (16, 27, 28). Furthermore, RAF has the potential to positively affect the risk of life-threatening crisis, alongside other morbidity and mortality factors.

In healthy individuals, pulsatile adrenocorticotropin secretion from the pituitary starts around 3 am and increases to a zenith around 7 am, accompanied by a concordant but delayed increase in adrenal cortisol secretion. As the day progresses, adrenocorticotropin pulse amplitude decreases, leading to reduced levels of serum cortisol by the late afternoon and evening: this circadian variation is the key to optimizing GC replacement in patients with Addison’s disease (4, 29). Exogenous steroid replacement cannot replicate this entirely, which is one of the key drivers for continuing to investigate methods of harnessing and exploiting endogenous steroidogenic capability. The presence of RAF and the fluctuating nature of endogenous steroidogenesis observed in this and previous studies reflects the heterogeneity of AAD. Previously, it was a widespread assumption that the instigation of exogenous steroids caused adrenal glands to entirely cease to function. It seems probable that adrenocorticotropin drive diminishes rapidly following the start of steroid replacement in most patients, compounding functional steroidogenic failure. However, these studies have accumulated evidence that intrinsic gland function can exist, even years after diagnosis, and has the potential to be exploited. Discovery of this heterogeneity is comparable to gains in knowledge in recent years of a spectrum of disease and a subset of patients with persisting C-peptide positivity indicative of a degree of maintained ß-cell function in type 1 diabetes. In both conditions, the disease trajectory within and between individuals is variable, resulting in greater scope for intervention to harness residual gland function and potentially improve patient outcomes—a further example of opportunity for the development of personalized medicine.

One significant challenge in detecting and monitoring RAF is the requirement for daily steroids in AAD. This limits the assessment of intrinsic gland function in a routine, outpatient clinical setting. The identification of a biomarker that could act as a surrogate for endogenous GC production would be particularly advantageous. Further attention should also be given to the choice of immunotherapy administered as rescue therapy in the setting of immune-mediated adrenocortical destruction. Rituximab was chosen in this early-phase study because of its mechanism of action, efficacy in similar diseases, partially successful outcome in our initial study (15), and its durable safety record over 2 decades. Although some participants experienced mild side effects during administration of the drug, it was essentially well tolerated—a key consideration in an experimental study using a novel therapeutic approach. Alternative immunotherapies, such as those that can maintain or enhance the activity of regulatory T cells, may prove more robust for tackling diseases with immune-mediated gland destruction, such as type 1 diabetes (30) and AAD, but are likely to be less well tolerated. Consequently, they may not be acceptable to patients considering participation in early-phase studies or panels considering ethical approval. Furthermore, it is evident from this study that once repeated adrenocorticotropin stimulation is withdrawn, its effect on adrenocortical steroid production rapidly wanes in most cases.

## Conclusion

Although harnessing and exploiting RAF remains a significant challenge, this experimental study has added further weight to the evidence that a sizeable proportion of patients with AAD have maintained endogenous steroidogenic potential after diagnosis. Our understanding of physiological steroid production means we know that standard steroid replacement is an imperfect therapy for patients with AAD—but what does detectable RAF or an improvement in endogenous function really mean for patients? There is a wealth of evidence that patients with the condition exhibit increased morbidity and reduced quality of life: Improved intrinsic gland function can be expected to counteract these problems to a degree, although the numbers in pilot clinical studies are too small to provide robust evidence of superior outcomes in the context of improving RAF. Nevertheless, there have been no trials of novel therapies other than alternative steroid replacement for AAD for more than half a century: persisting with innovative therapeutic approaches is the only meaningful prospect for delivering a tangible improvement in the lives of those with the condition.

## Abbreviations

21OH: 21-hydroxylase
AAD: autoimmune Addison disease
GC: glucocorticoids
MC: mineralocorticoids
RAF: residual adrenal function
SST: short Synacthen test

## Additional Information

***Disclosure Summary:*** The authors have nothing to disclose.

***Data Availability:*** The datasets generated during and/or analyzed during the current study are not publicly available but are available from the corresponding author on reasonable request.
